# Surgery

**Published:** 1876-09

**Authors:** 


### II. Surgery.

1. *Application of Caoutchouc in Surgery.* (*The Medical and Surgical Reporter*, May 27, 1876.)

M. Tarogenne, of Lyons, thus speaks of the cauterization of tissues rendered anæmic by means of Esmarch's elastic bandage:

The cauterization gives better results than when it is practiced on parts in which the circulation has not been suspended. Although it is not apparent at the moment of operation, the hot iron produces its effects more deeply. The surface for cauterization contains no liquid, it does not produce vapor, and the operator can watch the exact points to be attacked better. The integuments do not redden under the influence of the radiating caloric; they preserve their color or become slightly pale. The extent and depth of the cauterization are only shown when the elastic bandage is removed. These effects can be explained by the diminished loss of heat that the iron undergoes when it is not in contact with liquids, which, as a result of the high temperature, are converted into vapors. When it is necessary to act on fungoid tissues in osseous parts deeply situated, this means should be preferred to all others.

2. *Diagnostic Value of the Ileo-Femoral Triangle in cases of Injury of the Hip Joint; more particularly of Impacted Fracture.* Thomas Bryant, F.R.C.S. (*The London Lancet*, April, 1876.)

In a clinical lecture at Guy's Hospital Mr. Bryant cites a number of cases illustrating the value of the ileo-femoral triangle in the diagnosis of impacted fracture of the neck of the femur. The triangle is so called because it is found between the ilium and the femur. The lines which form it are, he claims, readily made out, and any shortening of the one which he terms the base, can be easily detected. One side of this right angled triangle is represented by a line drawn from the anterior superior spinous process of the ilium to the top of the trochanter major. The second is drawn from the anterior superior spinous process of the ilium directly downwards to the horizontal plane of the recumbent body. The third, which is the base of the triangle, is drawn at right angles to the second, and falls upon the first where it touches the great trochanter. It is to this third line the lecturer's observations refer, and he considers it the test line for fracture or shortening of the neck. Mr. Bryant confidently asserts, upon repeated proofs, that whilst in a healthy subject these ilio-femoral triangles are exactly similar upon the two sides of the body with equal sides and equal angles, with equal confidence assures us that in all cases of injury to the hip in which shortening of the neck of the thigh bone exists, the amount of shortening can readily and accurately be made out on comparing the vases of the triangles of the two sides. That in impacted fracture of the neck of the thigh bone, where on the sound side, the base of the triangle will, in the adult, measure its average normal of two and a half inches, on the affected or injured side, it will measure from half an inch to more than one inch less. The measurements should be taken with the patient in the horizontal position, the pelvis being at a right angle with the spine and the two femora parallel. A tape is then allowed to fall from the anterior superior spinous process of the ilium of one side to the horizontal plane of the body, and a second tape employed to measure the distance between this vertical tape and the upper border of the great trochanter on the same side. These are compared with measurements from corresponding points on the opposite side of the body. Under these circumstances there is no need for rough manipulation of the injured limb, no looking for crepitus, no forced flexion of the thigh, abduction or rotation.

3. *New Methods in Esmarch's Bloodless Operations.* (*The Medical Record*, New York, May 6, 1876.)

Prof. Esmarch published in Langenbeck's Archives some new applications of the elastic bandage for bloodless operations. The application was successfully used in a case of myxosarcoma of the right axilla, which required a removal of the whole arm, with the scapula, and the outer half of the clavicle. He found the plan very efficient in preventing hæmorrhage during an operation for the relief of a man almost the whole of whose penis, with the anterior surface of the scrotum, had been destroyed by epithelial cancer. In reply to the question how long the principal parts of the body may be kept in the bloodless condition, he said that the two lower extremities may be kept so completely for two hours and a quarter without injury.

4. *On Hospital Gangrene.* (*The Medical and Surgical Reporter*, April 15, 1876.)

In a paper in the Archiv für Klin. Chirurg., by Prof. Von Nussbaum, some details of much practical importance are given with regard to the prevention and curative treatment of this affection. In 1872, the first year of its appearance in the hospital, the gangrenous condition of the wounds in those attacked was always readily and successfully controlled by the local application of lotions containing nitrate of silver, corrosive sublimate, or carbolic acid; but as the distinctive changes became more and more acute, it was found necessary to have recourse to more active means, and to apply caustic paste and the actual cautery. Energetic applications of the latter agent proved the most efficacious, and a perfectly successful result of such treatment was usually indicated by a previous fall of the patient's temperature. During the prevalence of the gangrene many different attempts were made to protect healthy wounds and sores from contagion. The continuous water bath, applications of ice, moist warmth, lotions of carbolic acid, salicylic acid, chlorine water, etc., were tried without good results. Finally Lister's antiseptic plan of dressing was practiced most strictly, so that no open surface was dressed save under the carbolic acid spray, and no instruments or dressings used save after careful disinfection. Hospital gangrene at once ceased, and not a single case has been observed in Prof. Von Nussbaum's ward since the adoption of this plan of dressing, although at the period of its first use eighty per cent. of the surgical patients had been affected. He holds that the secret of Lister's method lay in the pedantic exactness in its mode of application.

5. *The Prevention of Pain after Applications of the Actual Cautery.* R. J. Levis, M.D. (*The Medieal Times*, Philadelphia, March 18, 1876.)

Dr. Levis makes use of the local anæsthetic action of carbolic acid in his frequent resort to the actual cautery, with the result of the most complete avoidance of consequent suffering. His practice is to apply pure carbolic acid on and for a short distance around each point of application of the cautery before the patient recovers from the influence of the general anæsthetic which has been used. The crystals of carbolic acid are deliquesced by warmth, and the liquid applied with a brush. The part is then covered with a light dressing. Should pain recur after extensive or deep use of the cautery, the application may be renewed. He has not, however, found a second application necessary.

